# Economic Consequences of the COVID-19 Outbreak: the Need for Epidemic Preparedness

**DOI:** 10.3389/fpubh.2020.00241

**Published:** 2020-05-29

**Authors:** Anton Pak, Oyelola A. Adegboye, Adeshina I. Adekunle, Kazi M. Rahman, Emma S. McBryde, Damon P. Eisen

**Affiliations:** ^1^Australian Institute of Tropical Health and Medicine, James Cook University, Townsville, QLD, Australia; ^2^North Coast Public Health Unit, New South Wales Health, Lismore, NSW, Australia; ^3^The University of Sydney, University Centre for Rural Health, Lismore, NSW, Australia

**Keywords:** SARS-CoV-2, COVID-19, global markets, economy, Coronavirus, pandemic

## Abstract

COVID-19 is not only a global pandemic and public health crisis; it has also severely affected the global economy and financial markets. Significant reductions in income, a rise in unemployment, and disruptions in the transportation, service, and manufacturing industries are among the consequences of the disease mitigation measures that have been implemented in many countries. It has become clear that most governments in the world underestimated the risks of rapid COVID-19 spread and were mostly reactive in their crisis response. As disease outbreaks are not likely to disappear in the near future, proactive international actions are required to not only save lives but also protect economic prosperity.

## Covid-19 and the Economy

On March 11, 2020, the World Health Organization (WHO) characterized COVID-19 as a pandemic, pointing to over 3 million cases and 207,973 deaths in 213 countries and territories (1). The infection has not only become a public health crisis but has also affected the global economy. Significant economic impact has already occurred across the globe due to reduced productivity, loss of life, business closures, trade disruption, and decimation of the tourism industry. COVID-19 may be that a “wake-up” call for global leaders to intensify cooperation on epidemic preparedness and provide the necessary financing for international collective action. There has been ample information on the expected economic and health costs of infectious disease outbreaks (2, 3), but the world has failed to adequately invest in preventive and preparedness measures to mitigate the risks of large epidemics.

**Figure 1:**
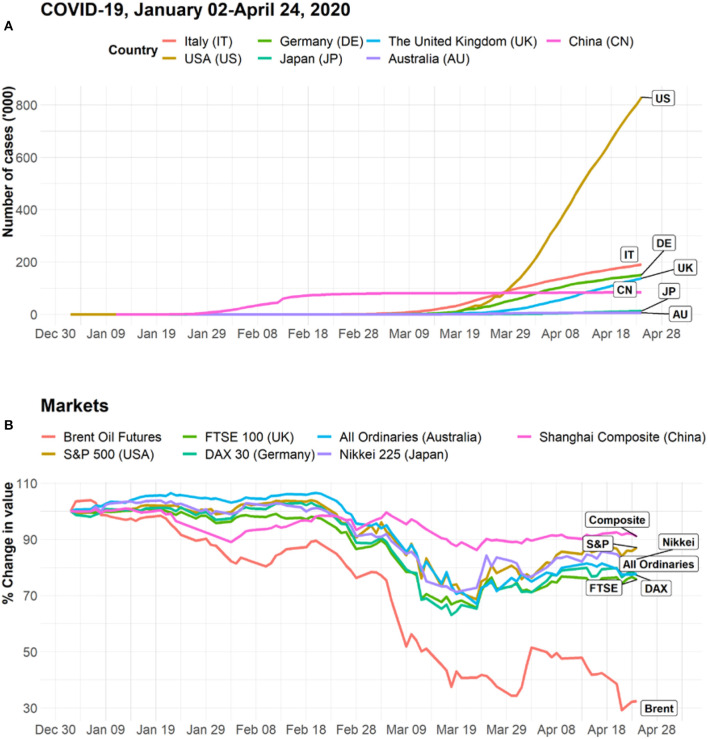
**(A)** Cumulative number of confirmed cases in emerging epicenters. Data sources: WHO Coronavirus disease (COVID-2019) situation reports (14). **(B)** Dynamics of the value of stock indices and oil futures relative to January 2, 2020. Data sources: historical data for stock indices and Brent oil futures were extracted from Yahoo Finance (www.finance.yahoo.com). Closing prices are used in the calculations. Daily values are calculated relative to an index value (100) on January 2, 2020.

With globalization, urbanization, and environmental change, infectious disease outbreaks and epidemics have become global threats requiring a collective response. Although the majority of developed countries, predominantly European and North American, have strong real-time surveillance and health systems to manage infectious disease spread, improvements in public health capacity in low-income and high-risk countries—including human and animal surveillance, workforce preparedness, and strengthening laboratory resources—need to be supported by using national resources supplemented with international donor funding. International collective action among governments, non-government organizations, and private companies has been advocated in building and financing technological platforms to accelerate the research on and development response to new pathogens with epidemic potential (2, 4). In the case of COVID-19, such cooperation is critical, especially for the development and production of a vaccine. The Coalition for Epidemic Preparedness Innovations (CEPI), a global partnership launched in 2017, has tracked global efforts in COVID-19 vaccine development activity and is advocating for strong international cooperation to ensure that vaccine, when developed, will be manufactured in sufficient quantities and that equitable access will be provided to all nations regardless of ability to pay (5). Furthermore, affected countries may benefit from exchanging technological innovations in contact tracing, such as health Quick Response (QR) codes, to manage the outbreak more effectively. However, there are important privacy implications that need to be considered (6). In the case of COVID-19, the collective response and adoption of preventive measures to stop the global spread were implemented too late, after COVID-19 had already penetrated other regions through international travel. Figure 1A presents the dynamics of confirmed COVID-19 cases and shows that large countries in Europe (e.g., Italy, Germany, and the UK) and the U.S. have already outnumbered China, the origin of epidemic, in the number of confirmed COVID-19 cases.

In addition to the substantial burden on healthcare systems, COVID-19 has had major economic consequences for the affected countries. The COVID-19 pandemic has caused direct impacts on income due to premature deaths, workplace absenteeism, and reduction in productivity and has created a negative supply shock, with manufacturing productive activity slowing down due to global supply chain disruptions and closures of factories. For example, in China, the production index in February declined by more than 54% from the preceding month's value (7). In addition to the impact on productive economic activities, consumers typically changed their spending behavior, mainly due to decreased income and household finances, as well as the fear and panic that accompany the epidemic. Service industries such as tourism, hospitality, and transportation have suffered significant losses due to reduction in travel. The International Air Transport Association projects a loss in airline revenue solely from passenger carriage of up to $314 billion (8). Restaurants and bars, travel and transportation, entertainment, and sensitive manufacturing are among the sectors in the U.S. that are the worst affected by the COVID-19 quarantine measures (9). The advance seasonally adjusted insured unemployment rate in the U.S. has already reached a record level of 11% for the week ending April 11, 2020 (10).

In addition to marked health inequalities, especially in countries without universal healthcare coverage, the economic impact of the COVID-19 pandemic will be heterogeneous across the country's income distribution. For example, office workers are more likely to transition to flexible working arrangements during the restrictions, while many industrial, tourism, retail, and transport workers will suffer a significant reduction in work due to community restrictions and low demand for their goods and services.

**Figure 2:**
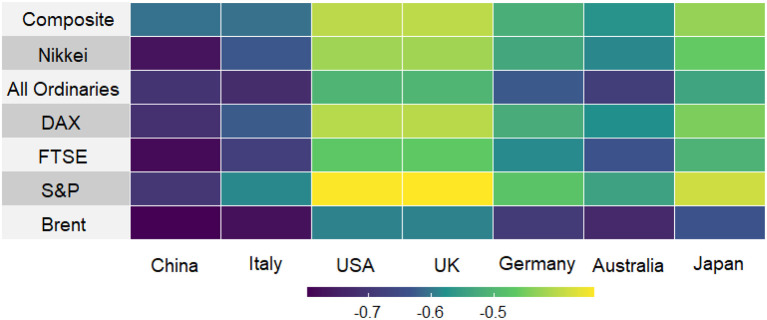
Correlation between the number of COVID-19 cases and stock markets in selected countries.

Global financial markets have been heavily impacted by the effects of COVID-19 spread. As the numbers of cases started to increase globally, mainly through the US, Italy, Spain, Germany, France, Iran, and South Korea, the world financial and oil markets significantly declined. Since the start of the year, leading U.S. and European stock market indices (the S&P 500, FTSE 100, CAC 40, and DAX) have lost a quarter of their value, with oil prices declining by more than 65% as of April 24, 2020 (Figure 1B). Daily data on stock market volatility and price movements are good indicators of consumer and business confidence in the economy. There were significant negative relationships between the daily number of COVID-19 cases and various stock indices (Figure 2). The correlation ranges from −0.34 to −0.80.

Larger economic problems are associated with the current and potential future demand for oil translating into fluctuations in oil prices due to reduced economic activities driven by the COVID-19 pandemic. Expected excess supply was also responsible for significant price reductions. If lower than expected oil prices continue, many oil-dependent economies may contract following reductions in trade and investment. Shocks to the labor markets will be severe, especially for countries dependent on migration. Globally, migrant workers make important contributions to the labor markets, addressing imbalances in both high- and low-skilled occupations (11, 12). As international travel restrictions and quarantine are likely to remain for the foreseeable future as countries try to halt the spread of COVID-19, migration flows will be limited, hindering global economic growth, and development (13).

## Conclusion

As the spread of the virus is likely to continue disrupting economic activity and negatively impact manufacturing and service industries, especially in developed countries, we expect that financial markets will continue to be volatile. There is still a question as to whether this unfolding crisis will have a lasting structural impact on the global economy or largely short-term financial and economic consequences. In either case, it is evident that communicable diseases such as COVID-19 have the potential to inflict severe economic and financial costs on regional and global economies. Because of high transportation connectivity, globalization, and economic interconnectedness, it has been extremely difficult and costly to contain the virus and mitigate the importation risks once the disease started to spread in multiple locations. This warrants international collective action and global investment in vaccine development and distribution, as well as preventive measures including capacity building in real-time surveillance and the development of contact tracing capabilities at the national and international levels. As outbreaks of novel infections are not likely to disappear in the near future, proactive international actions are required not only to save lives but also to protect economic prosperity.

## Author Contributions

AP and OA conceived and designed the study. AP and OA analyzed the data. AP, OA, AA, KR, EM, and DE contributed to the writing of the manuscript.

## Conflict of Interest

The authors declare that the research was conducted in the absence of any commercial or financial relationships that could be construed as a potential conflict of interest.
